# A New Elliptical Model for Device-Free Localization

**DOI:** 10.3390/s16040577

**Published:** 2016-04-22

**Authors:** Qian Lei, Haijian Zhang, Hong Sun, Linling Tang

**Affiliations:** 1 School of Electronic Information, Wuhan University, 430072 Wuhan, China; leiqian_0521@whu.edu.cn (Q.L.); hongsun@whu.edu.cn (H.S.); 2 State Key Laboratory of Information Engineering in Surveying, Mapping and Remote Sensing, Wuhan University, 430079 Wuhan, China; tanglinling@whu.edu.cn

**Keywords:** device-free localization, wireless sensor networks, radio tomographic imaging, elliptical model, orthogonal matching pursuit

## Abstract

Device-free localization (DFL) based on wireless sensor networks (WSNs) is expected to detect and locate a person without the need for any wireless devices. Radio tomographic imaging (RTI) has attracted wide attention from researchers as an emerging important technology in WSNs. However, there is much room for improvement in localization estimation accuracy. In this paper, we propose a geometry-based elliptical model and adopt the orthogonal matching pursuit (OMP) algorithm. The new elliptical model uses not only line-of-sight information, but also non-line-of-sight information, which divides one ellipse into several areas with different weights. Meanwhile the OMP, which can eliminate extra bright spots in image reconstruction, is used to derive an image estimator. The experimental results demonstrate that the proposed algorithm could improve the accuracy of positioning by up to 23.8% for one person and 33.3% for two persons over some state-of-the-art RTI methods.

## 1. Introduction

Device-free localization (DFL) [1] has attracted a great deal of research attention and is a significant technology in wireless sensor networks (WSNs) [2,3]. DFL is a technique crucial for detecting and tracking human bodies in indoor and outdoor environments without the need for any physical devices (e.g., sensors or tags) attached to them, which is useful for emergency or security personnel [4,5].

DFL uses three main measuring techniques: (1) ultrawideband (UWB) [5], (2) narrowband (NB) [6], and (3) received signal strength (RSS) [7,8,9,10,11]. As described in [5], UWB usually uses a nanosecond pulse to deliver information. UWB is a promising technology in DFL in unknown environments because of its low power consumption, low cost, high data rate, and high positioning accuracy. Because the center frequency of an NB signal is greater than its bandwidth, localization via signal delay is very difficult [6]. The RSS-based method has the advantages of lower cost, simple hardware, and lower power consumption.

There are six popular algorithms in DFL systems: (1) the fingerprint (FP) [12,13]; (2) the support vector machine (SVM) [14,15]; (3) radio tomographic imaging (RTI) [11,16,17,18,19]; (4) the particle filter (PF) [6,20]; (5) the Bayesian system [16,21]; and (6) compressed sensing (CS) [22,23]. Of these algorithms, the RTI-based DFL system has seen much research in recent years and is the focus of this paper.

As for RTI, the authors in [24] examined correlations between the communication links. Then, according to the principle of computed tomography (CT), for the first time an RTI algorithm was proposed by [18] that could reveal the position of a person’s body within the network area by imaging its attenuation.

With the development of DFL, RTI has attracted more and more research. A robust, low-cost Bayesian grid approach was proposed by [16]. Because of signal pollution and erroneous detection by wireless links, a novel nonlinear optimization approach with outlier link rejection for RSS-based DFL was proposed in [17]. As for RTI improvement, a new elliptical model could play a significant role in the accuracy of localization, as described in [19,25]. As described in [19], a measurement-based statistical elliptical model was proposed that could locate people in different environments. In [25], the voxel was the key factor in considering an elliptical model, instead of the distance between the sensors in each link, and a new elliptical model was proposed that was unlike existing RTI models. One of the problems with RTI was the ill-posed inverse. To solve this problem, Tikhonov regularization has been used for image reconstruction [18]. In addition, location accuracy was improved in another approach named regularized least-squares, adopted in deriving an image estimator, which had a good mean-square error (MSE) [19]. We have found that new elliptical models and solutions to the ill-posed inverse problem in RTI have become popular research areas.

In this paper, we propose a new, geometry-based elliptical model that improves location accuracy. In this model, ellipses representing communication links are divided into several different areas. Weightings are different in the different areas. In addition, the wireless channels inside one ellipse are divided into line-of-sight and non-line-of-sight paths, which is more consistent with the actual situation [26]. As for the ill-posed inverse, the orthogonal matching pursuit (OMP) algorithm is adopted for image reconstruction. The main contributions of this paper are the proposal of the new elliptical model and the adoption of the OMP to improve RTI localization accuracy.

This paper is organized as follows: In Section 2 background information about RTI is provided. In Section 3 we discuss the limitations of the RTI algorithm and introduce the new elliptical model. OMP for image reconstruction is explained in Section 4, and results are presented in Section 5. Finally, Section 6 draws conclusions.

## 2. Radio Tomographic Imaging

When entering a network area, a human body creates shadowing losses, which absorbs, diffracts, reflects, or scatters some of the power of the electromagnetic waves [18]. The reason for this is that the resonance frequency of water is 2.4 GHz, which most wireless sensor systems adopt, and 70% of the composition of a human body is water [27]. Furthermore, as described in [24], shadowing losses on different links are relevant. In other words, the most common cause of attenuation of different links would be human bodies.

**Figure 1:**
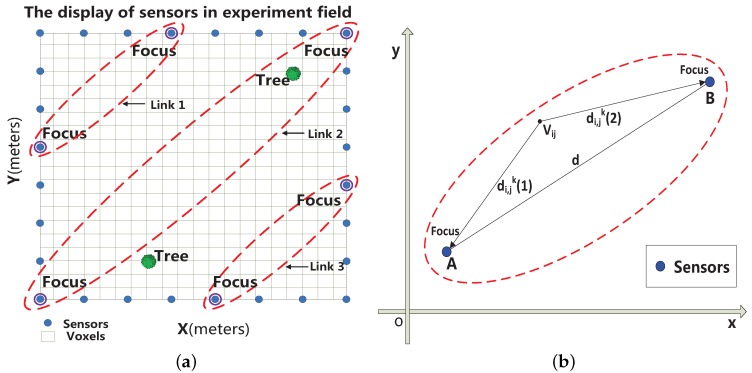
(**a**) The monitoring environment used in the experiments; (**b**) The elliptical model which is used in [18,19] representing one link in the monitoring area.

The objective of RTI is to locate a person without requiring them to wear or carry any electronic device, which may be useful in security breaches and emergencies [18,19]. The RTI system can be illustrated as in Figure 1a: *N* sensors are all around the monitoring area; the monitoring area can be divided into voxels, and the voxels can be described as a matrix whose dimensions are $M_{1}\times M_{2}$ . In Figure 1a two trees are in this area; the models of propagation of the electromagnetic waves are represented as ellipses, which represent links of communication [28]. Each “Focus”, which is a sensor, represents a focus of one ellipse. In [18], the change of each link in the RTI system is described in the following:

(1) $$\Delta y_{k}=\sum_{i=1}^{M_{1}}\sum_{j=1}^{M_{2}}W_{ij}^{k}\Delta x_{ij}+n_{k}$$

where *i* and *j* are the indices of voxels in the monitoring area, $i=1,2,3\ldots M_{1},j=1,2,3\ldots M_{2},M_{1}\in N_{+},M_{2}\in N_{+}$ ; *k* is the index of links, which are expressed as ellipses in the area; *K* is the number of links, $k=C_{N}^{2}=1,2,3\ldots K,K\in N_{+}$ ; $W_{ij}^{k}$ is the weighting of voxel $V_{ij}$ in link *k*; $\Delta x_{ij}$ is the attenuation change of voxel $V_{ij}$ ; $\Delta y_{k}$ is the change of signal power in link *k*; and $n_{k}$ is the noise of link *k*. The Equation (1) can be described in matrix form:

(2) Δy=WΔx+n

As described in [18], Δy , Δx , n and W can be defined in the following relationships:

(3) $$\Delta y={\left[\Delta y_{1},\Delta y_{2},\ldots ,\Delta y_{K}\right]}^{T}$$

(4) $$\Delta x={\left[\Delta x_{1},\Delta x_{2},\ldots ,\Delta x_{M_{1}\times M_{2}}\right]}^{T}$$

(5) $$n={\left[n_{1},n_{2},\ldots ,n_{K}\right]}^{T}$$

(6) $$W=\left[\begin{array}{cccc} W_{11} & W_{12} & \cdots & W_{1(M_{1}\times M_{2})}\\ W_{21} & W_{22} & \cdots & W_{2(M_{1}\times M_{2})}\\ \cdots & \cdots & \cdots & \cdots \\ W_{K1} & W_{i2} & \cdots & W_{K(M_{1}\times M_{2})} \end{array}\right]$$

where $M_{1}\times M_{2}$ is the number of voxels in the network, $M_{1}\in N_{+},M_{2}\in N_{+}$ ; Δy is the vector of signal power change in all links; Δx is the attenuation of all voxels; **n** is the vector of noise; **W** is the weighting model vector; and *T* represents transpose of a given matrix.

### 2.1. Elliptical Model

In [18], the elliptical model is described as:

(7) $$W_{ij}^{k}=\left\{\begin{array}{c} \frac{1}{\sqrt{d}}\;\text{if}\;d_{ij}^{k}\left(1\right)+d_{ij}^{k}\left(2\right)<d+\lambda \;\\ 0\;\;\;\;otherwise \end{array}\right.$$

where *d* is the distance between sensors *A* and *B* in link *k*, which is expressed as an ellipse in Figure 1b; *λ* is a parameter that determines the width of the ellipse; $d_{i,j}^{k}\left(1\right)$ is the distance between $V_{ij}$ and sensor *A* in link *k*; and $d_{i,j}^{k}\left(2\right)$ is the distance between voxel $V_{ij}$ and sensor *B* in link *k*.

### 2.2. Image Reconstruction

As for the image reconstruction, Δx estimated in Equation (2) is the ultimate aim of RTI. In general, the weighting matrix **W** is underdetermined, meaning that the same set of experiments can lead to multiple different images [19]. In other words, Δx estimated in Equation (2) is not unique, which is an ill-posed inverse problem. As presented in [18], the author used Tikhonov regularization:

(8) $$f\left(x\right)=\frac{1}{2}{∥W\Delta x-\Delta y∥}^{2}+\alpha \left({∥D_{x}x∥}^{2}+{∥D_{y}x∥}^{2}\right)$$

(9) $$\Delta x={\left(W^{T}W+\alpha \left(D_{x}^{T}D_{x}+D_{y}^{T}D_{y}\right)\right)}^{-1}W^{T}$$

where f(x) represents the objective function, $D_{x}$ and $D_{y}$ are the operators for horizontal and vertical directions, and *α* is the weighting parameter. In [19], another method of regularized least-squares was used in the solution to the ill-posed inverse problem.

## 3. Geometry-Based Elliptical Modeling

Existing methods for RTI concentrate mainly on elliptical models. To improve localization accuracy, many new elliptical models have been exploited by researchers. A new level-based, spatial elliptical model based on voxel links was proposed in [19]. However, there are some limitations to elliptical models: the weightings of voxels inside one link are the same [18,19], which is not consistent with the actual situation.

In fact, an ellipse, which represents the communication link in the monitoring area, can be divided into several areas. The weightings of voxels in different areas inside an ellipse should be different. In addition, communication channels inside one ellipse can be divided into line-of-sight paths and non-line-of-sight paths [26]. When a person stands on a line-of-sight path, the influence on the communication link is greater than when a person stands on a non-line-of-sight path inside the same weighting area. In addition, when the distance between a person and sensors is smaller, the interruption of the signal is greater. Hence, the elliptical model can be divided into different areas representing different weightings.

**Figure 2:**
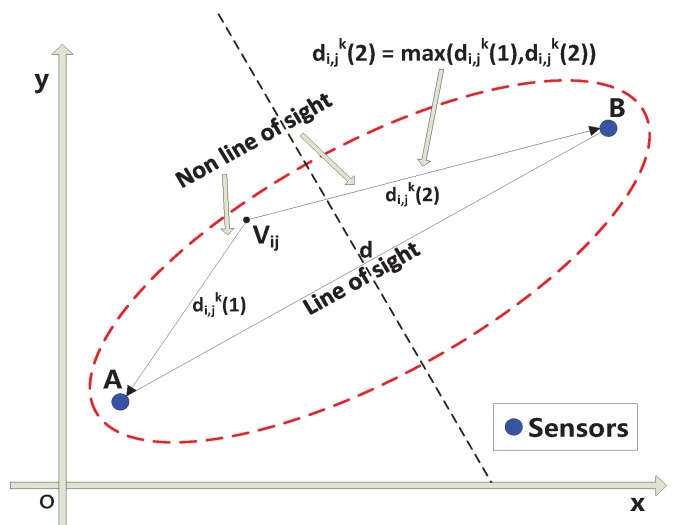
The communication channels inside one ellipse.

Based on the above discussion, the contribution of this paper is to propose a new elliptical model that concentrates on the insides of ellipses, which would be more in line with reality. As shown in Figure 2, the communication channels inside one ellipse can be grouped into two main categories: line-of-sight paths and non-line-of-sight paths [26]. $V_{ij}$ is the center of voxels in the ellipse. *A* and *B* are sensors; *i* and *j* are the indices of voxels, $i=1,2,3\ldots M_{1},j=1,2,3\ldots M_{2},M_{1}\in N_{+},M_{2}\in N_{+}$ ; *k* is the index of links, which are expressed as ellipses in the area; *d* is the distance between *A* and *B*; $d_{i,j}^{k}\left(1\right)$ is the distance between voxels *A* and $V_{ij}$ ; and $d_{i,j}^{k}\left(2\right)$ is the distance between *B* and $V_{ij}$ . A new elliptical model can be mathematically described as follows:

(10) $$W_{ij}^{k}=\left\{\begin{array}{c} \frac{1}{d}\left(k_{1}+max\left(d_{i,j}^{k}\left(1\right),d_{i,j}^{k}\left(2\right)\right)\right)\;\\ \;\;\;\;\text{if}\;d_{i,j}^{k}\left(1\right)+d_{i,j}^{k}\left(2\right)<d+\lambda ,\;d_{i,j}^{k}\left(1\right)+d_{i,j}^{k}\left(2\right)\neq d\;\\ \frac{1}{d}\left(k_{2}+max\left(d_{i,j}^{k}\left(1\right),d_{i,j}^{k}\left(2\right)\right)\right)\;\\ \;\;\;\;\text{if}\;d_{i,j}^{k}\left(1\right)+d_{i,j}^{k}\left(2\right)<d+\lambda ,\;d_{i,j}^{k}\left(1\right)+d_{i,j}^{k}\left(2\right)=d\;\\ 0\;\\ \;\;\;\;otherwise \end{array}\right.$$

where $k_{1}$ is a coefficient representing the obstacle to communication on the non-line-of-sight path, whose value is 2 by empirical experiments; $k_{2}$ is a coefficient representing the obstacle to communication on the line-of-sight path, whose value is 2.5 by empirical experiments. As for $d_{i,j}^{k}\left(1\right)$ and $d_{i,j}^{k}\left(2\right)$ , $max(d_{i,j}^{k}\left(1\right),d_{i,j}^{k}\left(2\right))$ represents the longer path. In Equation (10), the role of $max(d_{i,j}^{k}\left(1\right),d_{i,j}^{k}\left(2\right))$ is that it could divide one ellipse, which represents a communication link, into different areas. Moreover, weightings in different areas are different. We define $k_{2}=k_{1}+\beta$ , and *β* is a parameter. The relationships among $k_{1}$ , $k_{2}$ and *β* are described as follows, where the experiment settings are the same as in Section 5.

**Figure 3:**
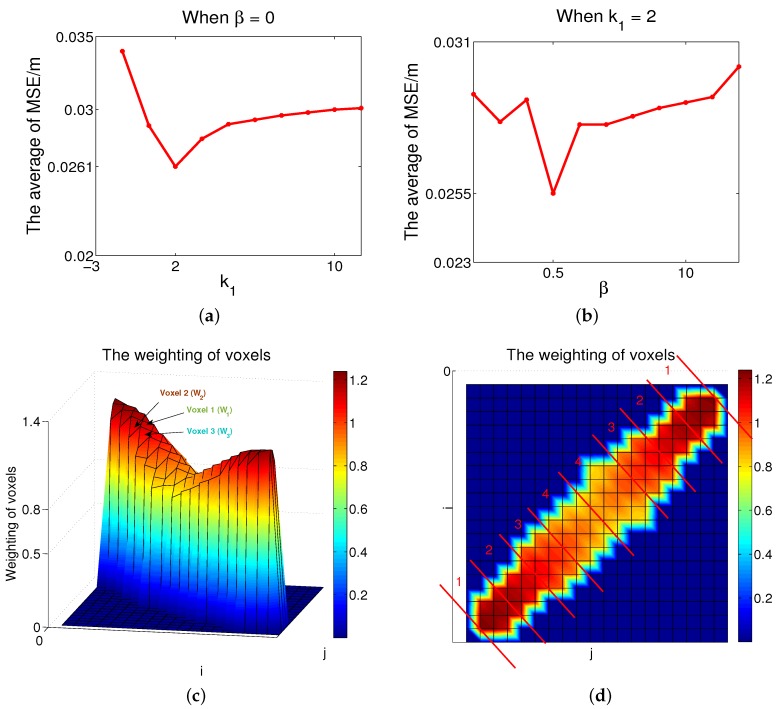
(**a**) When *β* = 0, the relationship between MSE and *k*_1_; (**b**) When *k*_1_ = 2, the relationship between mean-square error (MSE) and *β*; (**c**) The weightings of voxels, which is shown as link 2 in Figure 1a; (**d**) The division of the ellipse in Figure 3c.

To study the roles of $k_{1}$ and $k_{2}$ in Equation (10), first we set *β* as equal to zero. β=0 means there is no difference between line-of-sight paths and non-line-of-sight paths. As shown in Figure 3a, the vertical axis represents the average of MSE in all positions, and the horizontal axis shows the value of coefficient $k_{1}$ in Equation (10). From the results, when $k_{1}=2$ we achieve a better localization effect. In other words, $k_{1}=2$ is an optimization point. Second, we want to find the difference between the line-of-sight and non-line-of-sight paths. The optimization point of *β* needs to be found. To study the value of $k_{2}$ , we set $k_{1}$ as equal to 2. As shown in Figure 3b, β=0.5 is an optimization point. In other words, when $k_{2}=2.5$ , Equation (10) would achieve the best effect.

Link 2 in Figure 1a is the longest communication link. We can describe the weighting **W** of link 2 in Figure 3c and Figure 3d, where the experiment settings are the same as in Section 5. As shown in Figure 3c, the vertical axis represents the weighting of voxels; voxel 1 is on the line-of-sight path in the link, voxels 2 and 3 are on the non-line-of-sight path, $W_{1}$ represents the weighting of voxel 1, $W_{2}$ is the weighting of voxel 2, and $W_{3}$ is the weighting of voxel 3. It has been shown that $W_{1}$ was greater than $W_{2}$ , and $W_{2}$ was greater than $W_{3}$ . In other words, although voxel 2 was the closest to the nearest sensor in this link, the weighting of voxel 1 was greater than that of voxel 2. The reason is that voxel 1 was on the line-of-sight path, while voxel 2 was on the non-line-of-sight path. Although voxels 2 and 3 were both on the non-line-of-sight path, voxel 2 was closer to the nearest sensor in the same link. In addition, most voxel weightings were zero. The reason is that, compared to the number of voxels in the monitoring area the number of voxels inside a single link was small. As for the voxels whose weightings were not zero in the same link, the closer the voxels were to the sensors, the greater the values of weightings. It has been shown that voxel 2 was the closest to the nearest sensor in the link.

Furthermore, we can divide this ellipse, which is shown in Figure 3c, into several areas. As shown in Figure 3d, the ellipse, which represents the communication link, is divided into four areas. Voxels in different areas had different weightings. It has been shown that the values of weightings in area 1 were greater than those in areas 2, 3, and 4. The values of weightings in area 2 were greater than voxels in areas 3 and 4. Weightings of voxels in area 4 were smaller than those in other areas. The reason was that in area 1 the distances between the voxels and the sensor, which represented the focus of the ellipse, were smaller than those of voxels in other areas. In addition, the four areas (shown in Figure 3d) representing the weightings of voxels are shown in Table 1, which lists the ranges of the areas, where the experiment settings were the same as in Section 5. As a result, when voxels were on non-line-of-sight paths, the less the distances were between voxels and the nearest sensors in the same link, and the greater the weightings values.

## 4. Sparse-Based Image Reconstruction

New solutions to the ill-posed problem in RTI have become another way of improving localization accuracy. In [18], Tikhonov regularization was used for image reconstruction. In [19] an approach named regularized least-squares was adopted to derive an image estimator. The above methods had good results in localization. However, the number of bright spots, which represent the estimated positions, was greater than the number of persons in the monitoring area. Moreover, to some extent, the extra bright spots would increase the difficulty of localization.

In this paper, sparse-based algorithms, which can remove the extra bright spots in image reconstruction, are used in the solution to the ill-posed problem. Because compressed sensing can recover the original signal by sparse optimization, it draws a lot of attention from industry and academics [29]. The OMP, as a sparse representation algorithm, can recover a signal by finding optimal atoms in a sparse dictionary, which may be easier and faster to implement [30,31,32,33]. Localization accuracy can be improved at the same time. The procedure of the OMP algorithm used in image reconstruction can be written as:

**Step 1**  To initialize the counter of iteration t = 1, the set of index Λ = Φ, the residual $\Delta y_{r}=\Delta y$ .
**Step 2**  Pointer to the atom $i_{t}=\arg\max_{j=1,\ldots ,M}\left|\langle y_{r},\varphi_{j}\rangle \right|$ .
**Step 3**  To set the index $\Lambda =\Lambda \cup \left\{i_{t}\right\}$ .
**Step 4**  New estimation of signals $x_{r}=argmin{∥\Delta y-W_{\Lambda }\Delta y_{r}∥}_{2}$ .
**Step 5**  New residual signals $\Delta y_{r}=\Delta y-Wx_{r}$ , t=t+1 .
**Step 6**  If *t* ≥ *p*, loop will be terminated. If *t* < *p*, Step 2 will restart.

where *W* and Δ*y* are consistent with Equation (1); *p* is the sparsity representing the number of persons. When one person is in the system of network sensors, *p* can be set to 1, and there will be one bright spot in the image. Similarly, for two persons in the monitoring area *p* can be set to 2.

## 5. Experiment Results

### 5.1. Description of Experiment

The IEEE 802.15.4 communication standard was used, while the 2.4 GHz band was used for signal transmission. The MSE, which is used to measure the quality of algorithms, was defined as

(11) $$\epsilon =\frac{{∥x_{\text{real}}-x_{r}∥}^{2}}{M_{1}\times M_{2}}$$

where $x_{\text{real}}$ is the actual position for a person, $x_{r}$ is the estimated position by use of the proposed algorithm, and $M_{1}\times M_{2}$ is the number of voxels in the monitoring sensor area.

The measured data of the experiment were the same as in [18]. Compared to the system in [18], we used the same data set to derive the weighting parameters for the voxels before we used the same data again to localize. The improved weighting values in Equation (10) were related to $k_{1}$ and $k_{2}$ . The optimal values of $k_{1}$ and $k_{2}$ were empirically determined by measured data, which were shown in Figure 3a and Figure 3b. The experiment was done at the University of Utah, and the network was placed in an outdoor environment where there were 35 locations within the network, so that the persons’ locations were known for error analysis [18].

**Figure 4:**
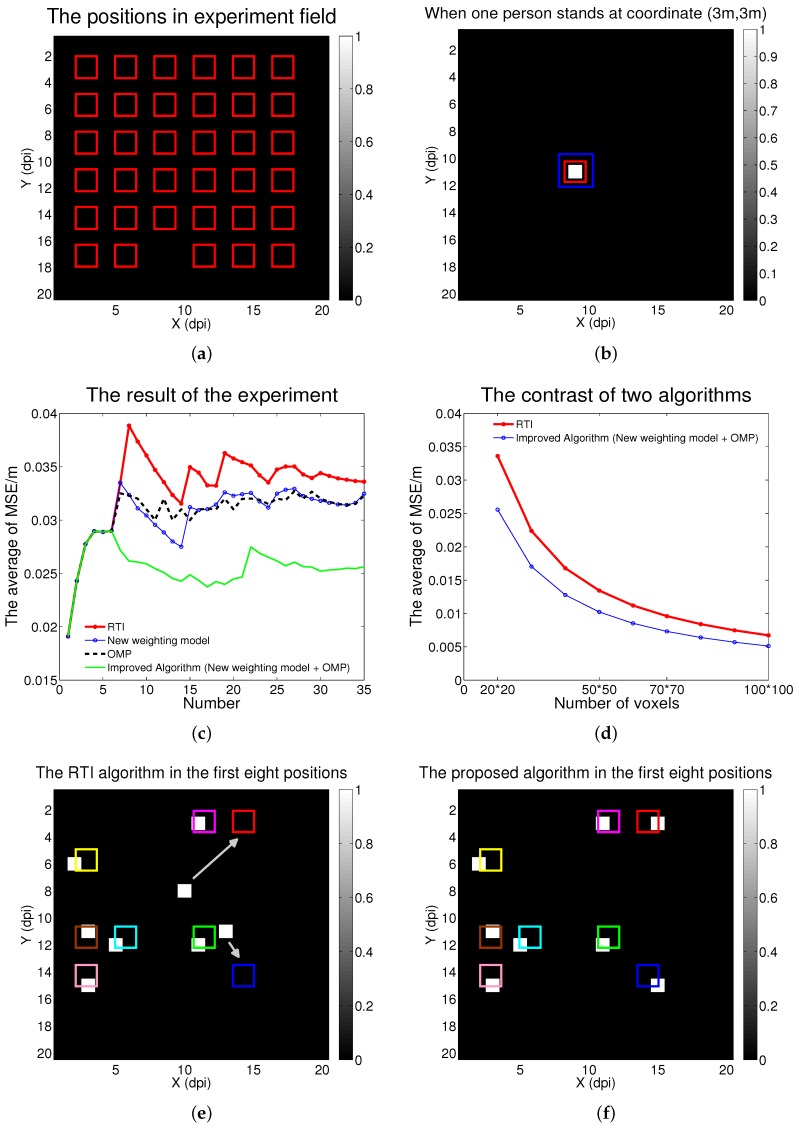
(**a**) All positions (red rectangles) in the experiment field; (**b**) The difference between the actual position (red rectangle), the estimated position (white voxel), and the experiment result in [18] (blue rectangle), when a person stood at coordinate (3 m,3 m); (**c**) The experiment result in all 35 positions for the localization of one person; (**d**) As the number of voxels increased, the contrast between two algorithms for the localization of one person; (**e**) The radio tomographic imaging (RTI) algorithm in the first eight positions for the localization of one person; (**f**) The proposed algorithm in the first eight positions for the localization of one person.

The monitoring area was 7 m × 7 m, and there were 35 positions to be located, which is shown in Figure 4a. There, red rectangles represent the positions for the localizations of one person or two persons. For the localization of one person, all 35 positions were used; *i.e.*, one person was located in every position. For the localization of two persons, six experiments were done. In other words, 12 positions were used at most. *N*, the number of sensors, was 28. $M_{1}$ and $M_{2}$ , the dimensions of voxels, were all 20. *K*, the number of links, was 378.

### 5.2. Experiment Result and Discussion

As show in Figure 4b, the red rectangle represents the actual position, the white voxel represents position estimated by the proposed algorithm, and the blue rectangle represents the experiment results in [18]. Compared to the RTI algorithm in [18], there were some advantages to the proposed algorithm. First, there were no extra bright spots that would affect the judgment of results during the process of image reconstruction, and the number of bright spots equaled the number of positions via the adjustment of sparse degrees. So the positions of persons could be confirmed as soon as possible. Second, the localization accuracy was improved, which is shown in the following experiment.

In Figure 4c, the horizontal axis shows the position numbers for one person, the vertical axis represents the average of the MSE, the red line displays the experiment results in [18], and the blue line displays the effect of using the proposed weighting model, with the method of Tikhonov regularization. The black points display the effect of using the OMP algorithm with the weight model in [18], and the cyan line represents the advantages of the improved algorithm. As the number of positions increased there was more noise, which came from fading loss, shadowing loss, and measurement noise in the experiment, which affected localization accuracy [18]. Hence, in the first five or six positions the MSE averages increased in the four algorithms. With a further increase in positions, the rise in the noise would be less than the increase in the number of positions because of the function of the proposed algorithm. So the MSE averages decreased in the next 8 or 10 positions. Finally, the increase in the noise, the increase in the number of positions, and the function of the algorithm were in a dynamic balance. The averages of MSE in the four algorithms tended toward stability. It was found that the proposed algorithm functioned the best of the four algorithms.

When the value of the horizontal axis in Figure 4c was eight, eight positions were selected randomly to be located for one person. As shown in Figure 4e and Figure 4f, the accuracy was better than state-of-the-art RTI methods. Compared to the RTI in [18], the proposed algorithm had greater localization accuracy, while the average of MSE in all places was reduced approximately 23.8%, which is shown in Figure 4c. The average of MSE for the first eight places was reduced approximately 32.6%, as shown in Figure 4e and Figure 4f.

In Figure 4d, the vertical axis shows the average of MSE in all 35 positions, the horizontal axis represents the number of voxels in the monitoring area, the red line shows the experiment results in [18], and the blue line shows the effect of the proposed algorithm. As the number of voxels increased, the average of MSE in all 35 positions between the two algorithms decreased. Furthermore, compared to state-of-the-art RTI methods, the proposed algorithm achieved a better localization effect. For the localization of two persons, the experiment was done as follows.

**Figure 5:**
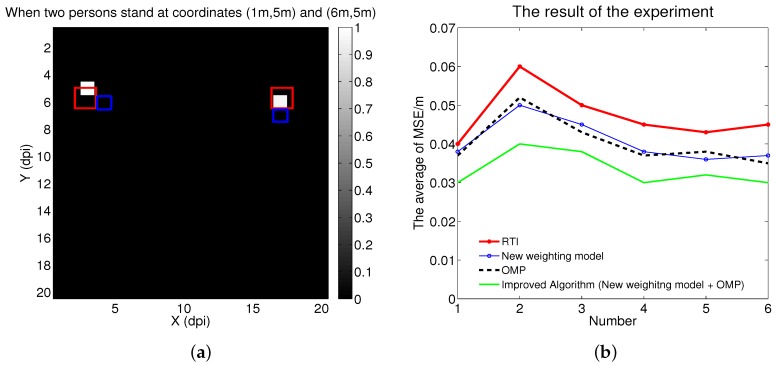
(**a**) The differences between the actual positions (red rectangles), the estimated positions (white voxels), and the experiment results in [18] (blue rectangles), when two persons stood at coordinates (1 m,5 m) and (6 m,5 m); (**b**) The result of the experiment for the localizations of two persons.

As shown in Figure 5a, the white voxels represent the positions estimated by the proposed algorithm, the red rectangles represent the actual positions, and the blue rectangles represent the experiment results in [18]. Compared to the RTI algorithm in [18], the accuracy of the localization was improved. For all six experiments for localizing two persons, the result is shown in Figure 5b.

As shown in Figure 5b, the horizontal axis shows the six experiments, the vertical axis represents the average of the MSE; the red line displays the experiment results in [18]; the black points display the effect of using the OMP algorithm, with the weight model in [18]; the blue line displays the effect of using the proposed weight model, with the method of Tikhonov regularization; and the cyan line represents the advantages of the proposed algorithm. Compared to the localization of one person, the two-person accuracy was slightly low. The reason is that as the number of persons increased, more noise came from fading loss, shadowing loss, and measurement noise, affecting accuracy [18]. As shown in Figure 5b, the effect of the proposed algorithm was better than that of the experiment results in [18]. In addition, the experiment results showed that the proposed algorithm could improve the accuracy of positioning up to 33.3% compared to the state-of-the-art RTI method.

In addition, for the image reconstruction we adapted the OMP algorithm, which improved the accuracy, as shown in Figure 4c and Figure 5b. However, we needed to know the number of persons beforehand, and RTI systems have no such limitation. In the future we will research ways to ameliorate this limitation.

## 6. Conclusions

In this paper, we propose a new weight model and adopt OMP for image reconstruction to enhance the accuracy of DFL. The new model concerns the insides of ellipses, which would be more in accordance with reality. And as a sparse representation algorithm, OMP dealt with the ill-posed inverse problem while preventing other bright spots in the image, which achieved a better accuracy.

In future work, on the one hand we will research a weight model with different geometry-based methods, which would promote the development of state-of-the-art RTI. On the other hand, more sparse-based methods could be utilized for image reconstruction.

## Figures and Tables

**Table 1:** The division of link 2 in Figure 1a.

Areas	The Range of Weightings

1	[1.12, 1.25]
2	[0.97, 1.08]
3	[0.75, 0.93]
4	[0.53, 0.70]

## Abbreviations

The following abbreviations are used in this manuscript:

DFL: Device-free localization
WSN: Wireless sensor network
RTI: Radio tomographic imaging
OMP: Orthogonal matching pursuit
UWB: Ultra-wideband
NB: Narrow-band
RSS: Received signal strength
FP: Fingerprint
SVM: Support vector machine
PF: Particle filter
CS: Compressed sensing
CT: Computed tomography
NOOLR: Nonlinear optimization approach with outlier link rejection
MSE: Mean-square error
